# A comprehensive dragon fruit image dataset for detecting the maturity and quality grading of dragon fruit

**DOI:** 10.1016/j.dib.2023.109936

**Published:** 2023-12-10

**Authors:** Tania Khatun, Md. Asraful Sharker Nirob, Prayma Bishshash, Morium Akter, Mohammad Shorif Uddin

**Affiliations:** a Department of Computer Science and Engineering, Daffodil International University, Dhaka, Bangladesh; b Department of Computer Science and Engineering, Jahangirnagar University, Dhaka, Bangladesh

**Keywords:** Dragon dataset, Image recognition, Agriculture, Deep learning, Computer vision

## Abstract

Dragon fruit, often referred to as pitaya, is a tropical fruit with various types, including both white-fleshed and red-fleshed varieties. Its distinctive appearance is complemented by a range of potential health advantages. These include its abundance of nutrients and antioxidants, which contribute to a robust immune system, aid in blood sugar regulation, and support the well-being of the heart, bones, and skin. Consequently, the global desire for dragon fruit is yielding substantial economic advantages for developing nations like Bangladesh, which in turn underscores the pressing need for an automated system to identify the optimal harvest time and differentiate between fresh and defective fruits to ensure quality. To accomplish this objective, this paper introduces an extensive collection of high-resolution dragon fruits because effective detection by machine learning models necessitates a substantial amount of data. The dataset was painstakingly gathered during a span of four months from three distinct locations in Bangladesh, with the valuable assistance of domain experts. Possible application of the dataset encompasses quality evaluation, robotic harvesting, and packaging systems, ultimately boosting the effectiveness of dragon fruit production procedures. The dataset has the potential to be a valuable resource for researchers interested in dragon fruit cultivation, offering a solid foundation for the application of computer vision and deep learning methods in the agricultural industry.

Specifications Table

**Table:** 

Subject	Computer Science
Specific subject area	Image Categorization, Image Detection, Robotic Harvesting, Maturity Analysis
Data format	Raw jpg
Type of data	Image
Data collection	In collaboration with an expert from Ministry of Agriculture, Bangladesh, a collection of images was taken during the period from May 2023 to August 2023 from the demonstration areas of three different locations in Bangladesh.
Data source location	**Location:** Bappi Taj Agro Farm demonstration farm in Gazipur, Tipu Sultan Agro Farm in Jhenaidah, and the Daffodil research farm in Gazaria, Munshiganj **Zone:** Gazipur, Jhenaidah, Munshiganj **Country:** Bangladesh
Data accessibility	**Repository name:** Mendeley Data **Data identification number**: 10.17632/2jpzbx8tm6.1 **Direct URL to data:**https://data.mendeley.com/datasets/2jpzbx8tm6/1

## 1 Value of the Data

• Inconsistent human harvesting practices lead to the risk of overripe or underripe fruit. Harvesting dragon fruit prematurely leads to decreased sweetness, flavor, and overall quality, potentially dissatisfying customers and reducing demand and sales, resulting in financial losses, increased labor costs, and lower prices for growers. Physical characteristics like the weight, texture, and external color of the peel are commonly employed as non-invasive techniques for assessing the ripeness of dragon fruit [1]. Therefore, utilizing the computer vision approach this dataset has the potential to develop an automated harvesting system that can empower farmers by delivering accurate advice on optimal harvest times by analyzing images of various fruit development stages, consequently lowering labor requirements and minimizing financial losses.
• Detecting the freshness and identifying defects in dragon fruit is essential for upholding product quality, minimizing wastage, avoiding economic repercussions, as well as creating avenues for international exports while promoting the production of top-tier goods to satisfy global market requirements [2]. The dragon fruit image dataset presented in this article can play a pivotal role in this endeavor by serving as an asset for training computer vision and deep learning models. This involvement aids in quality assurance, waste reduction, optimized harvesting practices, and the automation of inspection processes. Ultimately, the dataset's application results in improved product quality, economic advantages for growers and the agricultural sector, and heightened customer contentment, underscoring its significance in fresh and defective dragon fruit detection.
• This dragon fruit dataset is significant for researchers as it serves as a valuable resource for developing and testing computer vision, machine learning, and deep learning technologies. Researchers can create automated systems for fruit recognition, improving harvest efficiency, predicting freshness, and automating packaging and this dataset encourages interdisciplinary collaboration between computer scientists and experts in other fields, particularly agriculture. Moreover, the dataset has the potential to deliver economic benefits by reducing labor costs and enhancing crop quality, underscoring its relevance and importance in the field of computer science.

## 2 Background

The compilation of this dataset arose out of the need to address challenges in identifying dragon fruit developmental stages prevalent in agriculture. The creation of the dataset aligns with ongoing efforts in precision agriculture, which aims to improve crop management practices through technological interventions. Motivation also arose from the lack of comprehensive datasets specific to dragon fruit stages and diseases, which hindered the development of accurate detection models. We collect 3780 images displaying different growth stages, and conditions, this dataset serves as a valuable resource for training and validating deep learning algorithms and enables fast and accurate detection of dragon fruit stages and qualities. The dataset article complements a related research publication by providing researchers and practitioners with access to raw data, increasing transparency, reproducibility, and further investigations to optimize agricultural practices.

## 3 Data Description

The dataset comprises images that depict different phases of dragon fruit development, encompassing healthy young fruits, ripe fruits, and decayed specimens. These images were manually taken during the period spanning from May to August 2023 from the demonstration farm of Bappy Taz Agro Farm in Gazipur, Tipu Sultan Agro Farm in Jhenaidah, and the Daffodil Research Farm in Gazaria, Munshigonj with guidance from a domain expert using the cameras of a Redmi Note 11 Pro Plus and a Samsung S22 smartphone. The resultant images with sizes 800×800 pixels are captured and stored in the JPG format. Each image in the dataset is labeled according to its corresponding stage of maturity and quality, allowing for easy classification and analysis.

While gathering pictures from the dragon fruit orchard, we ran into a few difficulties such as,

1. The primary challenge encountered during data collection pertained to capturing images amidst noisy backgrounds and uneven lighting conditions.
2. The growth of dragon fruits is very time-sensitive. For the dataset to be accurate and relevant, photos had to be collected at growth stages or during particular seasons.

**Fig. 1:**
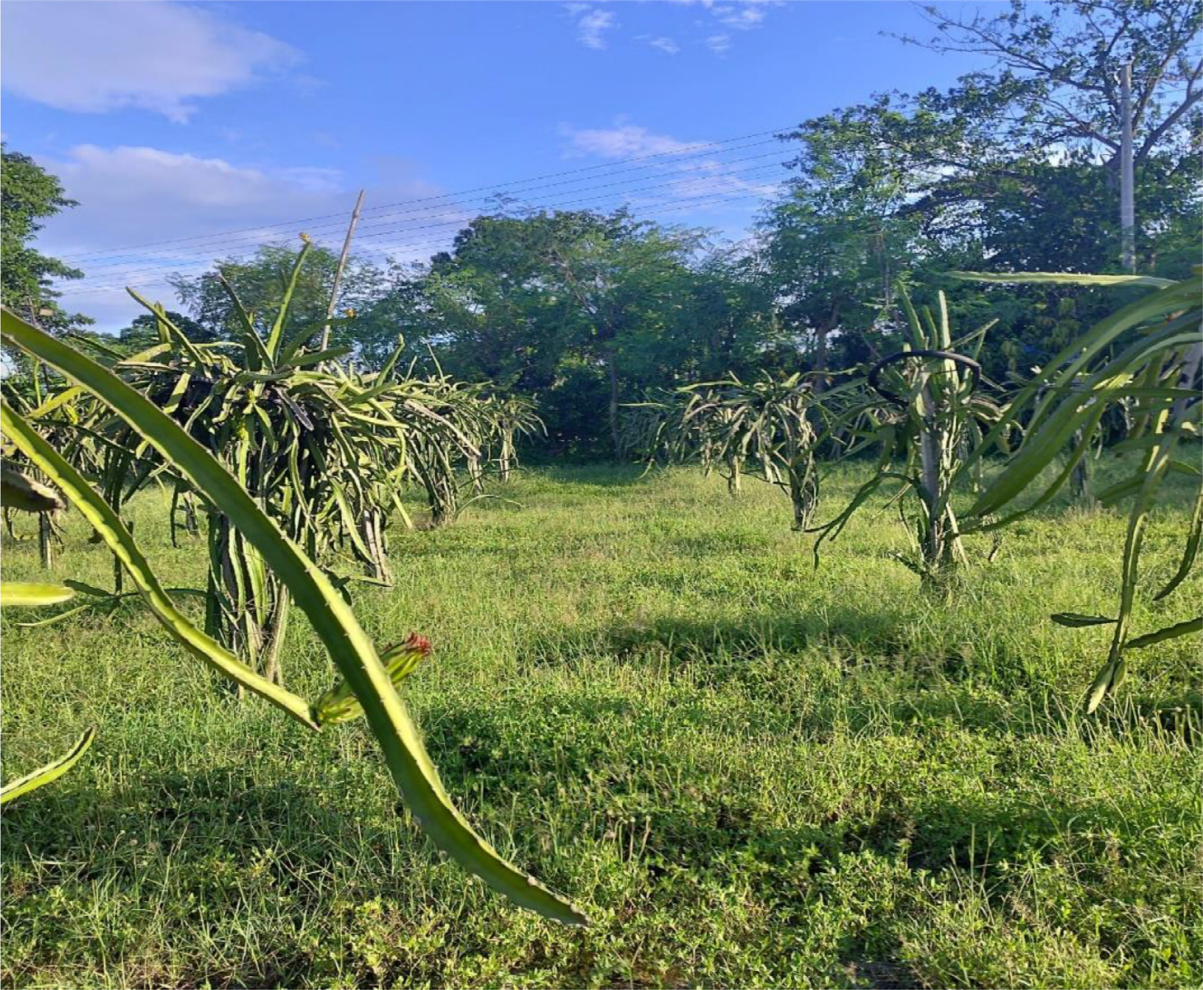
The real dragon fruit field from where we collected the dataset images.

Fig. 1 illustrates the dragon fruit field from where we gathered dataset images.

In this paper we have presented three varieties Bari Dragon Fruit-1, Connie Mayer Dragon Fruit, and Thai Red Dragon Fruit. Table 1 represents the details of these varieties of dragon fruits.

**Table 1:** Details about the dragon fruit varieties in the dataset.

Variety Name	Description	Visualization
Bari Dragon Fruit-1	Bari Dragon Fruit-1, a red dragon fruit variant, was invented by BARI (Bangladesh Agricultural Research Institute) and is now successfully cultivated in Dhaka, Chattagram, Northern areas of Bangladesh, and Rangamati Hill. At maturity, the fruit weighs around 350-400 grams and features a light pink exterior, revealing a dark pink, succulent interior boasting a Total Soluble Solids (TSS) content of 13.22%. With an edible portion comprising 81% of the fruit, this variety demonstrates consistent high-yield fruit production. A 3–5-year-old tree yields approximately 3.22 kg/year. Notably, it contains 12.06 millimicrograms of beta-carotene and 41.27 milligrams of vitamin C per 100 grams, highlighting its nutritional value [3].	
Connie Mayer Dragon Fruit	The Connie Mayer Dragon Fruit, has medium-sized blooms with inner petals shifting from purple to light pink edges, retaining an alluring appearance even during budding. These small, abundant fruits, weighing between 7 and 9 ounces, undergo a striking transformation as their green skin ripens into a transparent pink shade with lemon-lime green fins, enveloping sweet, white flesh. Notably, an extended vine-ripening time of around 45 days in Louisiana, compared to the standard 30 days for similar Hylocereus varieties, augments sweetness [4].	
Thai Red Dragon Fruit	Thai dragon fruit also referred to as pitaya, hails from Thailand and is part of the cactus family. It exists in diverse varieties, such as the white-fleshed Hylocereus Undatus and the red-fleshed Hylocereus Costaricensis or Hylocereus Polyrhizus types. Classified by the National Bureau of Agricultural Commodity and Food Standards under the Ministry of Agriculture and Cooperatives of Thailand, this fruit is divided into 3 primary groups based on skin and inner pulp colors [5]. Typically, these fruits are oval or elongated, featuring shiny, spiky skin. Renowned for its gently sweet taste, it's commonly relished in smoothies, akin to kiwi, and often used as an eye-catching garnish in culinary dishes due to its vivid appearance.	

In the field of agriculture science, Automation is a game-changer that benefits a nation's agriculture economy in several ways. The raising of quality is one of the main benefits. A final result that is uniform and of high quality is made possible by automation in tasks like fruit and vegetable sorting and grading. This is crucial for satisfying customer demands and those of global markets, which frequently have high standards for quality. While manual fruit and vegetable sorting is still common, it is well known to have a number of disadvantages. As human perception can be subjective and impacted by things like exhaustion or personal judgment, it is prone to mistakes and inconsistencies. Additionally, it takes a lot of time, especially when processing greater amounts of fruit, which can result in inefficiencies and higher labor expenses. Moreover, hand sorting can be expensive because Intelligent fruit grading systems have been created to address these issues. These systems use computer vision algorithms to classify and evaluate products automatically according to a variety of quality criteria. Computer vision makes it possible to precisely measure and analyze traits including color, texture, size, shape, and flaws.

To enable these advancements, this paper introduces two sets of data. The first dataset, referred to as the Dragon Fruit Maturity Detection Dataset, and the second dataset, the Dragon Fruit Quality Grading Dataset, are presented. Each of these dataset folders is further divided into two subfolders: the original dataset, consisting of images directly captured with a camera, and the augmented dataset, containing images generated from the original dataset using data augmentation software. The Dragon Fruit Maturity Detection Dataset takes up 976MB of space, while the Dragon Fruit Quality Grading Dataset occupies 624MB in its folder.

**Fig 2:**
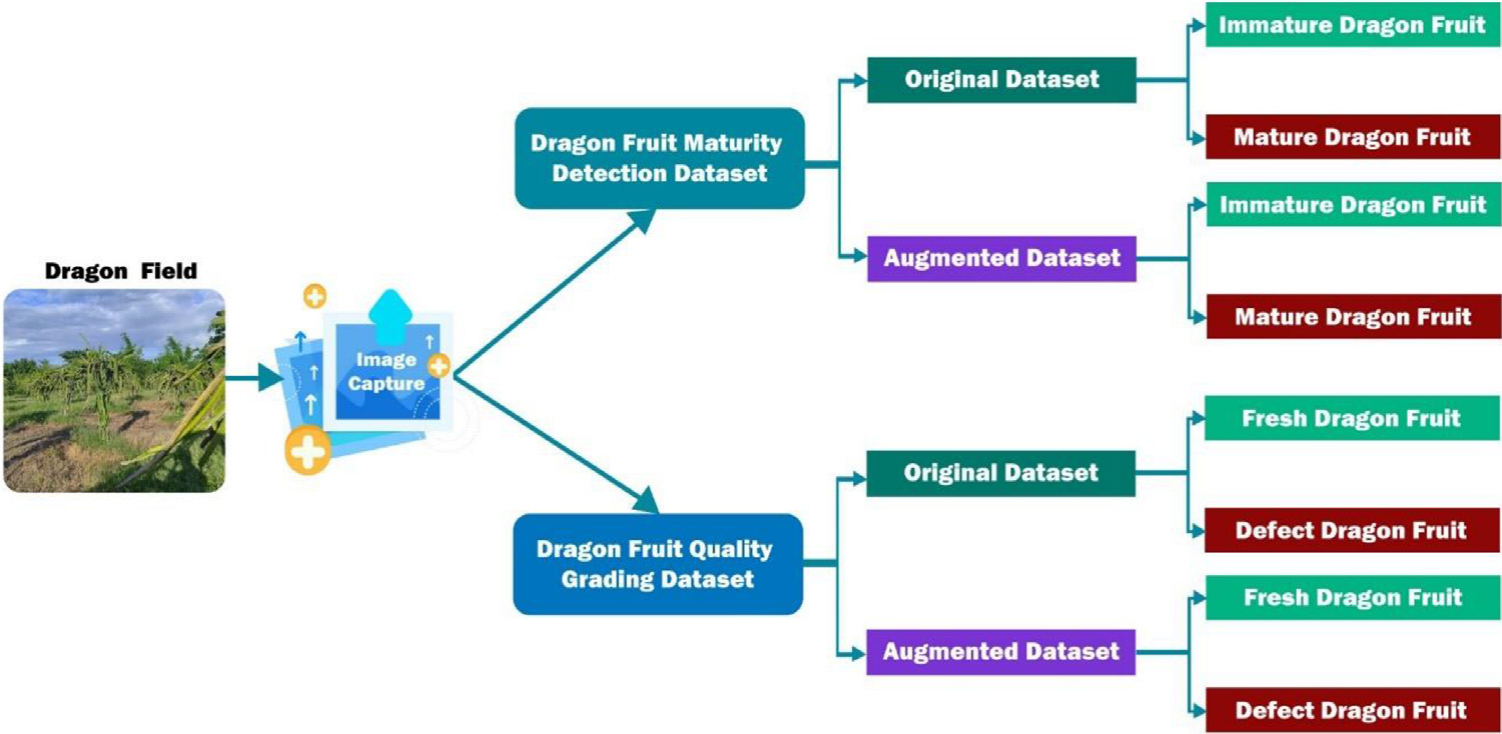
Organization of dragon fruit dataset.

The ripeness and quality of dragon fruits are closely linked to characteristics such as color, skin appearance, texture, flavor, size, and shape [1]. Within the Dragon Fruit Maturity Detection Dataset, both the original and augmented datasets are categorized into two groups: Mature Dragon Fruit and Immature Dragon Fruit. Similarly, within the Dragon Fruit Quality Grading Dataset, both the original and augmented datasets are divided into two groups: Fresh Dragon Fruit and Defect Dragon Fruit. Each of these folders includes relevant images of dragon fruits. The organization of the dataset is presented in Fig 2.

The progression of dragon fruit growth differs based on factors such as its variety, cultivation conditions, and climatic influences. Generally, it spans an average duration of 31 to 41 days, roughly equivalent to one and a half months, for the fruit to attain its full mature size [1]. Furthermore, it is important to harvest the fruit at its optimal stage of maturity to ensure the best quality, flavor, and texture [11]. Table 2 explains each category in both the Dragon Fruit Maturity Detection and Quality Grading Dataset.

**Table 2:** Concise overview of the dragon fruit maturity detection and quality grading dataset.

Topic	Class Name	Description	Visualization
Dragon Fruit Maturity Detection Dataset	Immature Dragon Fruit	Premature dragon fruit, in contrast to its ripe counterpart, is smaller in size, typically green, or light pink, has a firmer texture, a milder and less sweet flavor, underdeveloped seeds, and may exhibit a slightly sour taste [1]. Its firmness sets it apart from the softer and sweeter qualities of fully ripe dragon fruit. The exact characteristics can vary depending on the dragon fruit variety and its specific stage of ripeness.	
	Mature Dragon Fruit	A mature dragon fruit has a visually striking appearance. Mature dragon fruit is characterized by its larger size, vibrant red or magenta color based on variety, firm, and spiky skin, sweet and mildly tangy flavor, well-developed seeds, and a sweet tropical aroma when ripe. The skin is usually covered in scales or spikes, giving it a unique and exotic look [6].	
Dragon Fruit Quality Grading Dataset	Fresh Dragon Fruit	Depending on the variety, fresh dragon fruit has a bright exterior skin in tones of pink, red, or yellow. The skin typically has scales or spikes covering it, giving it an unusual and exotic appearance. The flesh can be white or red and is soft, juicy, and slightly crunchy due to small black seeds [7]. A vivid color, a subtle softness to the touch, and a delightful perfume are indications of ripeness.	
	Defect Dragon Fruit	One of the fruit's skin's changing look, becoming loose and wrinkled, is one of the early signs of spoiling. Additionally, these characteristics include physical damage, rot, over-ripeness, internal issues, the possibility of being hollow or empty, physical color changes, and moving from its typical pink hue to a purple one [8]. The interior of spoiled dragon fruit turns a deeper shade of brown.	

The dragon fruit dataset holds promise across various applications:

**Developing robotic harvesting systems:** The dragon fruit dataset serves as a pivotal resource in crafting sophisticated robotic harvesting systems capable of selectively picking ripe fruits through image analysis. Leveraging this dataset, machine learning models are trained to precisely locate and discern ripe dragon fruits amidst varying backgrounds. These models enable the development of algorithms that empower robots to make real-time decisions based on color, texture, and shape analysis, selectively harvesting only ripe fruits while leaving others to mature further. Moreover, integrating this dataset-derived intelligence into robotic systems not only streamlines fruit picking but also facilitates continuous learning and adaptation, refining the system's accuracy and efficiency in the dynamic context of fruit harvesting.

**Automating quality control processes:** The dragon fruit image dataset holds immense potential in automating quality control processes within packaging facilities. Through machine learning, this dataset can train models to assess various quality parameters, such as size, shape, color, and defects, enabling automated inspection of dragon fruits as they move through the packaging line. By leveraging the dataset, these systems can accurately identify, and sort fruits based on predetermined quality standards, ensuring consistency and adherence to quality benchmarks. Moreover, the dataset facilitates continuous learning, allowing the system to adapt and improve its accuracy over time, enhancing efficiency and precision in the packaging process while reducing human intervention.

## 4 Experimental Design, Materials and Methods

### 4.1 Camera specification

The information was collected by employing the cameras of a Redmi Note 11 Pro Plus and a Samsung S22 smartphone.

The camera of the Redmi Note 11 Pro Plus device is equipped with a 108MP Samsung ISOCELL HM2 sensor, which is a relatively large sensor with a size of 1/1.52 inches. The individual pixels on the sensor have a size of 0.7µm, but they can be combined using a technique called 9-in-1 binning, where 9 pixels are merged to create a larger pixel with a size of 2.1µm.

The Samsung S22 device's camera is furnished with a 50MP Samsung GN5 sensor and Sony IMX766 sensors, featuring a relatively spacious 1/1.57-inch sensor size. The individual pixels on this sensor measure 1.0 µm each, accompanied by an f/1.8 aperture.

### 4.2 Data augmentation

Data augmentation is essential for deep learning models, particularly for visual object recognition. It is a potent technique for strengthening deep learning models, in particular, supplements the training dataset by creating new images from the ones that already exist, enhancing model generalization, and reducing overfitting. We used a variety of augmentation strategies such as shearing, random rotation, horizontal flipping, width, and height changing, zooming, and brightness modifications. To increase the dataset's diversity and resilience, several procedures were used in accordance with accepted best practices.

The photos may be oriented in a variety of ways according to these specifications, which include a rotation range of 45 degrees. Additionally, we added a 0.2 width and height shift range, allowing for the displacement of the image's content in both directions. The controlled deformation was introduced with a shear range of 0.2. We changed the scale of the photographs by applying a zoom range of 0.2 to provide more diversity. The dataset was expanded with mirrored versions of the photos when horizontal flipping was enabled. We used the 'reflect' fill mode to manage picture modifications without any hiccups. Additionally, to ensure a dynamic range of lighting circumstances, we changed brightness in the range of 0.5 to 1.5. The robust and varied dataset produced by these parameter settings improved the deep learning models' training process.

Within our dataset, a code-driven, automated augmentation procedure was used to create a total of 10010 augmented pictures. These enhanced pictures were carefully designed to increase the variety and depth of our dataset. These improved images are skillfully paired with the appropriate original sample images for each category in Table 3. This careful pairing serves to give a clear and instructive representation of the results of the augmentation, successfully demonstrating the effectiveness of the data augmentation process in growing and enhancing our dataset.

**Table 3:** Statistics of the dragon fruit dataset.

Topic	Name of Class	Original Image Number	Augmented Image Number
Maturity Detection	Immature Dragon Fruit	1241	3000
	Mature Dragon Fruit	887	2010
Quality Grading	Fresh Dragon Fruit	898	2000
	Defect Dragon Fruit	754	3000
**Total**		**3779**	**10010**

**Fig. 3:**
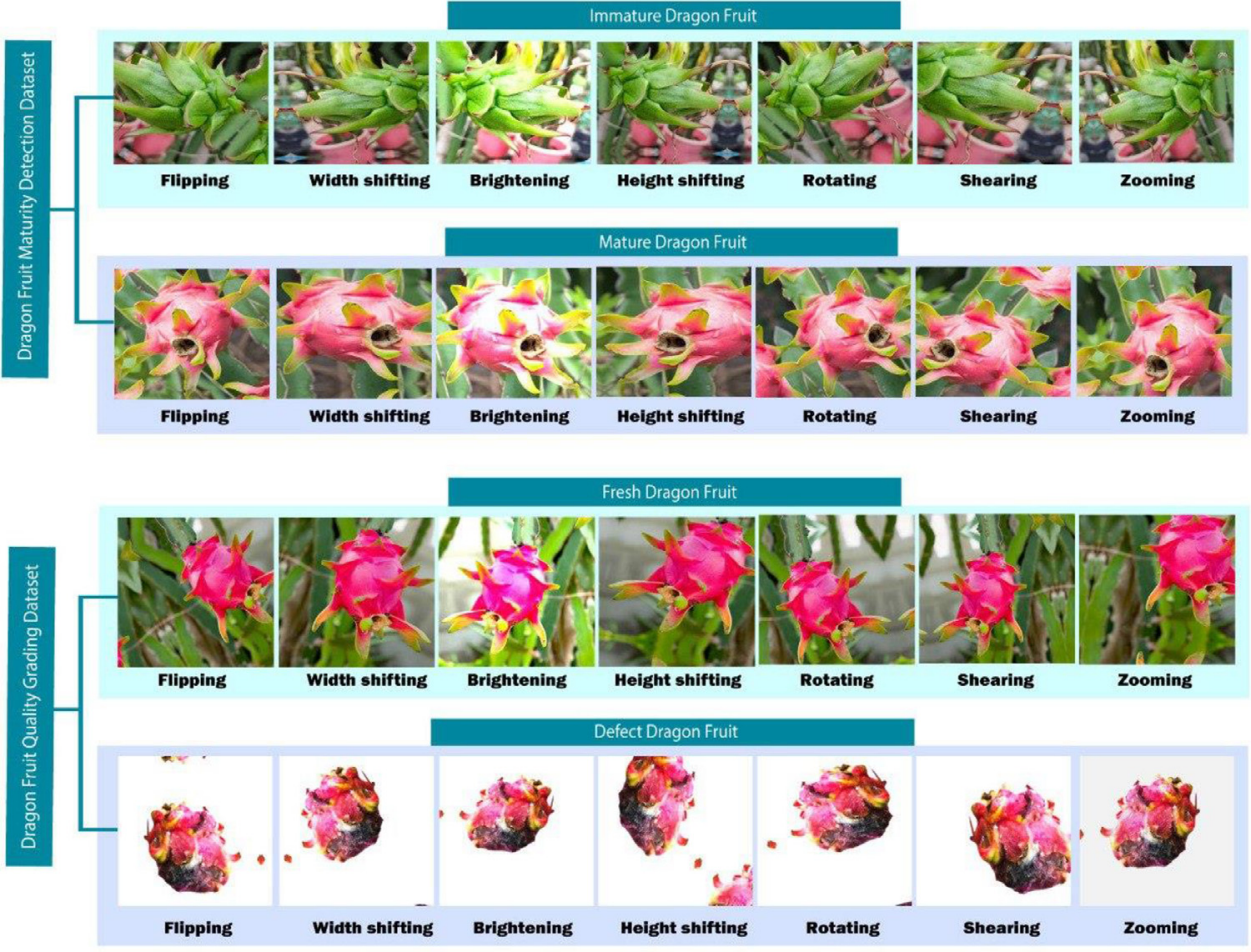
Augmented images of dragon fruit dataset.

The training dataset is given controlled variance, which makes the model more adaptable to actual-world circumstances. Our main methods include shearing for various viewpoints, horizontal/vertical shifting (up to 20% width/height), and random rotation (0-45 degrees). While horizontal flipping teaches orientation invariance, random zooming (80–120%) aids in managing various scales. A fill mode keeps the image's original content while adjusting the brightness (50–150%) and contrast (70–130%) to account for changes in lighting. Pre-processing adds a random zoom function, increasing the model's versatility. Models are now able to distinguish things in a variety of real-world scenarios thanks to these strategies. Fig. 3 displays the augmented images of dragon fruit from the dataset, while Table 3 provides the dataset's statistical information.

### 4.3 Deep learning model validation

We introduced a deep learning model designed to effectively train the dataset, striving for state-of-the-art outcomes. The validation of this deep learning model requires a thorough evaluation of its performance on a dataset. A deep learning model comprises interconnected layers of nodes, where each node signifies a computational unit. The input layer's nodes receive data, while the output layer's nodes generate the ultimate outcome. Situated between these input and output layers are hidden layers, housing the neural network's primary computational capacity [9]. Deep learning models have made substantial strides in analyzing visual data, including tasks such as classifying images or videos, detecting objects, and processing natural language [10]. The deep learning model follows a structured five-step process, encompassing data preprocessing, data segmentation, model training, performance evaluation on a validation set, and ultimately, testing the model on a completely distinct test set. This rigorous approach is crucial to verify the model's reliability in producing accurate results and its capacity to adapt to new data.

Data pre-processing is critical for deep learning because it prepares visual data for model input, enhances data quality, and influences model performance, generalization, and efficiency. It ensures that the images are in a suitable format for the computer vision tasks, addresses issues that can affect model learning and decision-making, and ultimately leads to more accurate and reliable results in various applications. In this research work, image pre-processing involves a range of data transformations, including actions such as data labeling, image resizing, image augmentation, and segmentation.

**Data labeling:** During the first round of data pre-processing, we scrupulously labeled the data, properly assigning each image to its corresponding class or category. Labeled data serves as the foundation for training and refining deep learning models; without precise labels, models are unable to acquire knowledge and make reliable predictions.

**Image resizing:** Because images within the dataset may come in different sizes, we found it necessary to resize them according to our specifications to provide a consistent and understandable dataset. This reduced computational demands and guaranteed compatibility during the training of deep learning models.

**Image segmentation:** As needed, we carried out image cropping to remove undesirable background elements, thereby improving the dataset's overall quality.

**Data augmentation:** The deep learning model requires a huge volume of data as it enhances model performance reduces overfitting and enables complex feature extraction [12]. Hence, we expand the dataset size by employing various augmentation techniques, as comprehensively outlined in Section 3.2.

**Fig. 4:**
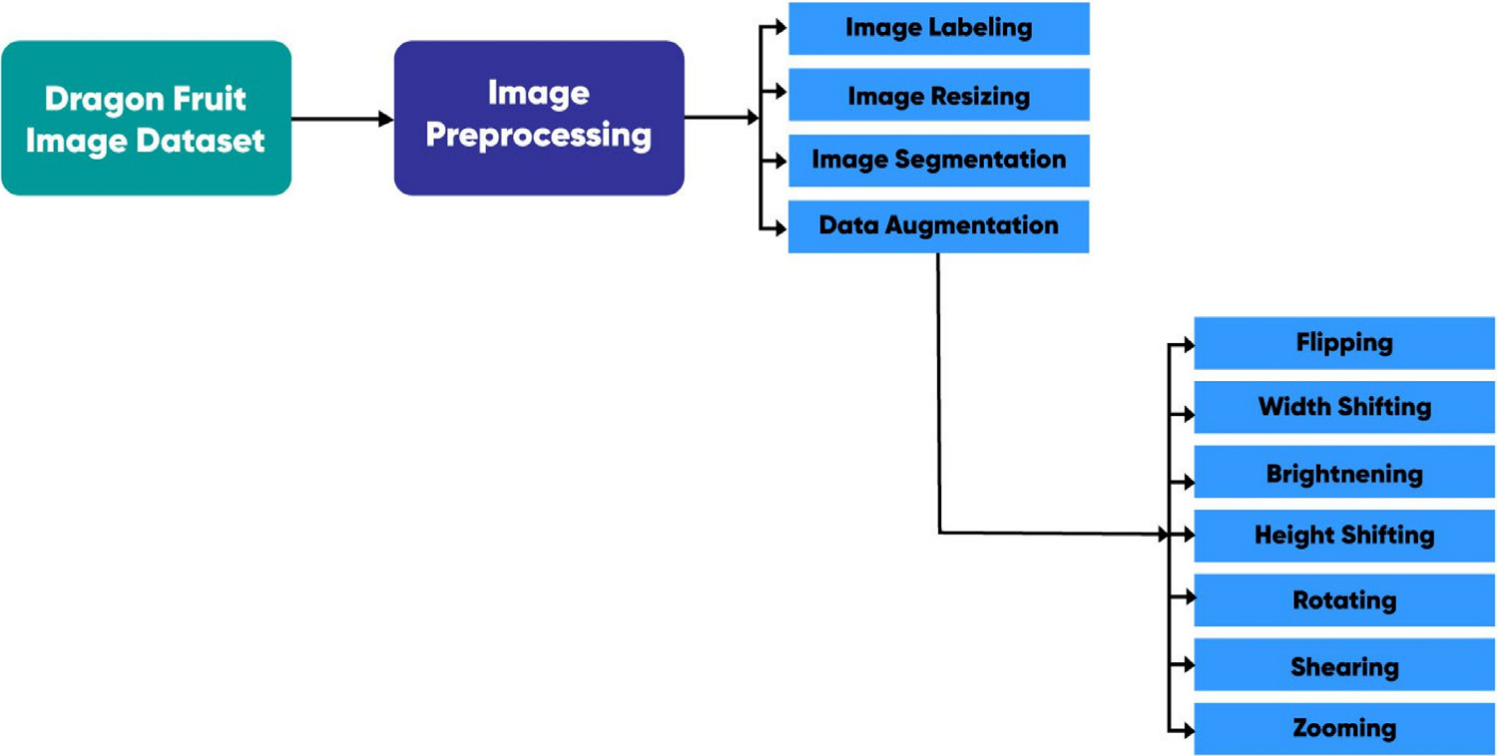
The pre-processing steps of proposed deep learning model.

Fig. 4 represents the pre-processing steps that we have applied to the dataset.

The dataset underwent a meticulous division into two distinct sets, namely the training dataset and the testing dataset, following a thoughtful separation process. This involved randomly selecting 80% of the photos to compose the training dataset, with the remaining 20% constituting the test dataset. Importantly, there were no repeated images shared between the training and test sets. The testing set played a pivotal role in evaluating the model's performance, serving as a robust benchmark after it had been trained on the training data.

**Fig. 5:**
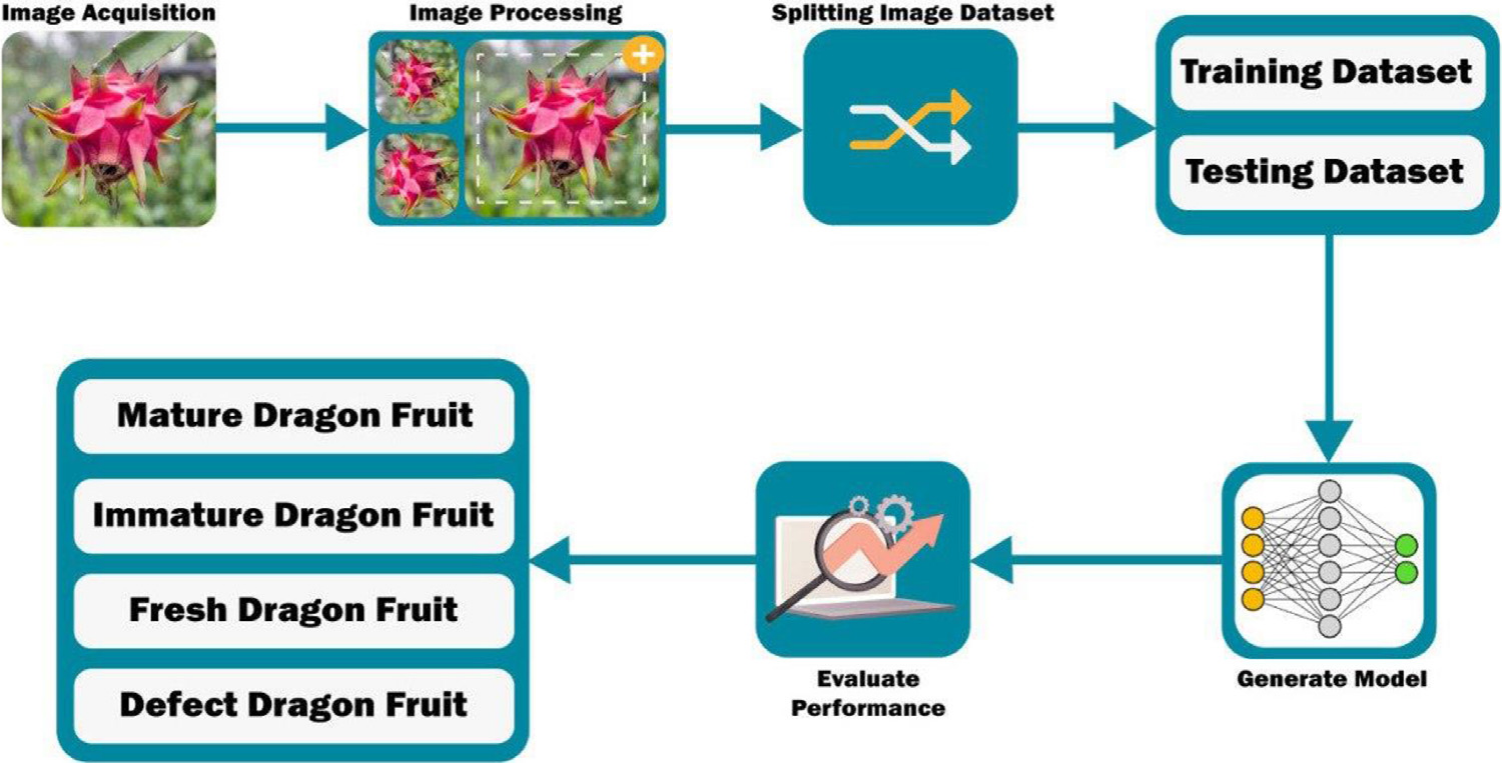
The working process for assessing dragon fruit ripeness and distinguishing between fresh and defective dragon fruits.

A comprehensive overview of the rigorous validation techniques employed in our deep-learning model, utilizing the dragon fruit image dataset, is thoughtfully presented in Fig. 5. These validation procedures encompassed various tasks, including the discrimination of mature and immature dragon fruit, as well as the classification of dragon fruit as fresh or defective. This validation framework ensured the model's effectiveness and reliability in achieving the specific objectives of our study.

#### 4.3.1 Model description

In this research, we have applied the ResNet50 framework with the intention of identifying the ripeness and quality of dragon fruits. ResNet50 is a commonly employed deep convolutional neural network (CNN) architecture that is renowned for its effectiveness in image classification and object detection [13]. It is characterized by its depth, consisting of 50 convolutional layers, which enables it to learn intricate features from images. Its deep structure and residual connections contribute to its ability to achieve state-of-the-art results in a variety of computer vision applications.

Within the ResNet-50 architecture, two types of blocks are present: the Identity Block and the Convolutional Block, where "identity block" is a specific type of residual block used within the architecture. Residual blocks are the main advancement in ResNet50, enabling the network to learn complex data representations by integrating shortcut connections and limiting overfitting by bypassing some layers. The result of the residual block is subsequently transferred to the following block. Convolutional blocks facilitate feature extraction and boost network performance with convolutional layers, batch normalization, and ReLU activation functions.

In ResNet50, batch normalization is applied after each convolutional layer and before the activation function (e.g., ReLU), ensuring that the inputs to subsequent layers are well-scaled and centered. ReLU introduces non-linearity into the network by replacing negative values with zeros. In addition, to reduce the spatial resolution, capturing the most important information while reducing computational complexity max pooling layer is used periodically which involves selecting the maximum value in a local region of the feature map.

The architecture concludes with a global average pooling layer, followed by a fully connected layer and softmax layer. ResNet employs global average pooling as an alternative to the conventional fully connected layers, which serves to decrease spatial dimensions and create a feature vector. The classification output is generated through a last fully connected layer, and the quantity of neurons within this layer is determined by the number of categories involved in the classification task. The softmax layer in ResNet-50 serves the purpose of converting the raw output into a probability distribution, particularly for multi-class classification tasks. It ensures that the network's output represents the likelihood of the input belonging to different classes, making it easier to determine the predicted class and calculate the loss during training.

#### 4.3.2 Measurement metrics

In the context of deep learning and classification tasks, an assessment matrix that incorporates metrics like Accuracy, Precision, Recall, and F1-Score is frequently utilized. These indicators are crucial for evaluating the effectiveness of categorization model performance. Here is a quick breakdown of each metric:

**Accuracy:** A classification model's accuracy serves as a gauge of its general correctness. Instances properly predicted as a percentage of all instances in the dataset are calculated. Although accuracy is a valuable indicator, it may not give a whole view of model performance, particularly when working with datasets that are unbalanced.

(1) $$Accuracy=\frac{True\;Positive+True\;Negative}{True\;Positive+True\;Negative+False\;Positive+False\;Negative}$$

Precision: Precision indicates how accurately the model's optimistic predictions came true. It measures the proportion of real positives to all anticipated positives.

(2) $$Precision=\frac{True\;Positive}{True\;Positive+False\;Positive}$$

**Recall:** The model's capacity to recognize all pertinent instances in the dataset is measured by recall, also known as sensitivity or true positive rate. It measures the proportion of real positives to all actual positives.

(3) $$Recall=\frac{True\;Positive}{True\;Positive+False\;Negative}$$

**F1-Score:** The harmonic mean of recall and precision is known as the F1-Score. When you need to take into account both false positives and false negatives, it provides a balance between these two measures and is particularly helpful.

(4) $$F1-Score=2\times \frac{Recall\;\times Precision}{Recall\;\pm Precision}$$

**Fig. 6:**
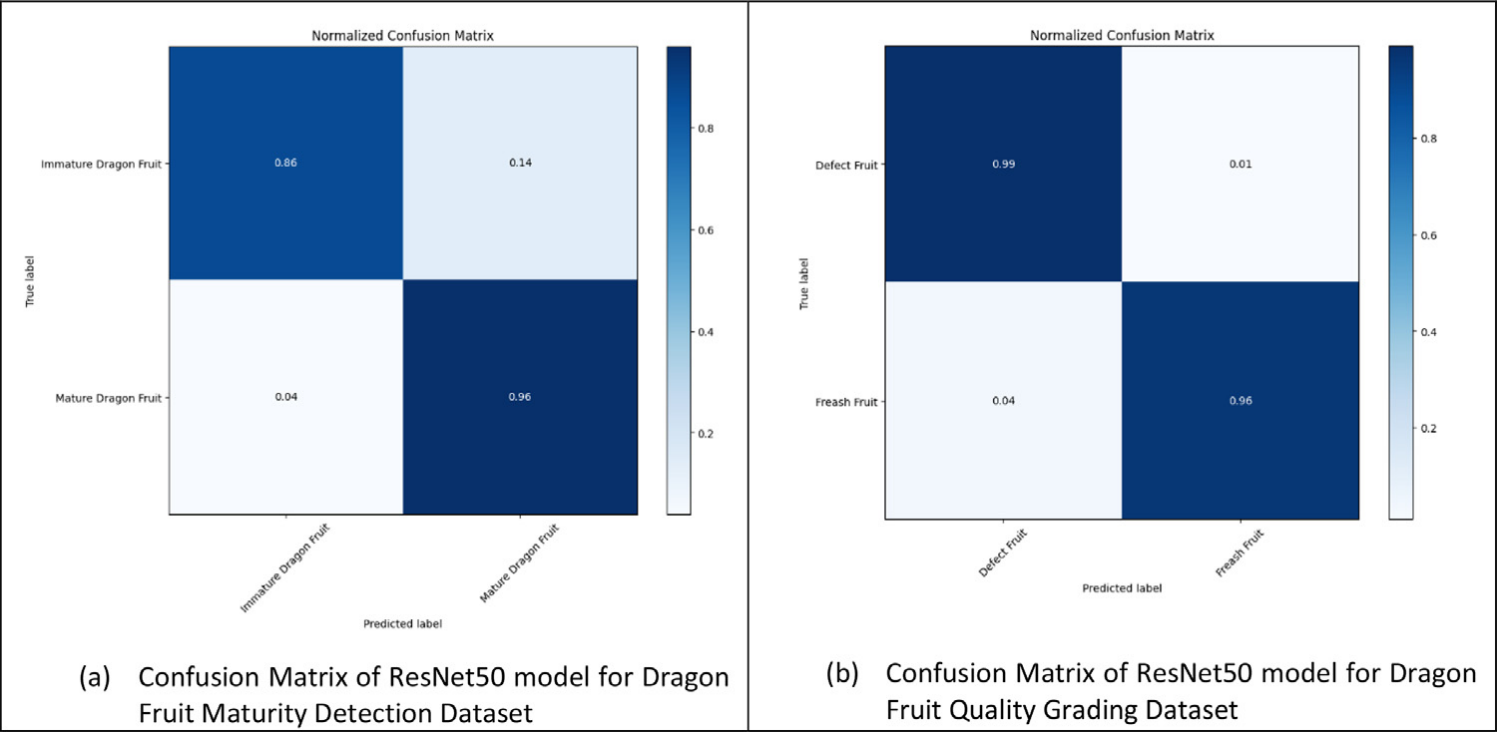
Confusion matrix of ResNet50 model.

**Confusion matrix:** A crucial tool for assessing the effectiveness of classification models, particularly in situations with several classes, is the confusion matrix. It gives a thorough understanding of how closely the model's predictions match the actual class labels for distinct categories. This matrix is crucial for identifying the model's benefits and drawbacks when categorizing various groups, allowing for a thorough assessment of its effectiveness. The confusion matrix equips data scientists to make well-informed decisions, comprehend class-specific performance, and pinpoint areas for development by classifying forecasts into true positives, true negatives, false positives, and false negatives. The following Fig. 6 represents the confusion matrix of ResNet50 model for dragon fruit maturity detection and quality grading dataset.

This amazing achievement underlines the ResNet50 architecture's potency in precisely determining the maturity and quality grading of dragons. The model has demonstrated strong performance, achieving a 90% accuracy rate in distinguishing between immature and mature dragon fruit and a 98% accuracy rate in identifying fresh or damaged dragon fruit which is clearly depicted in Table 4. This outstanding performance highlights the model's strong ability to generalize to new data, demonstrating its utility for real-world applications.

**Table 4:** Classification report for maturity detection and quality grading.

Topic	Class Name	Precision	Recall	F1-Score
Dragon Fruit Maturity Detection Dataset	Immature Dragon fruit	0.97	0.86	0.91
	Mature Dragon fruit	0.82	0.96	0.89
	Accuracy			0.90
Dragon Fruit Quality Grading Dataset	Defect Fruit	0.97	0.99	0.98
	Fresh Fruit	0.98	0.96	0.97
	Accuracy			0.98

In the times to come, we will thoroughly investigate advanced deep learning models with the help of this dataset to identify the most effective approach for real-world applications. In the future, by utilizing machine learning algorithms and AI for image processing we will develop a consumer-oriented mobile app aiding in selecting ripe, fresh, and defective dragon fruits.

## Limitations

The classification of any other fruit would not be possible for this dataset because it solely relates to and is primarily focused on dragon fruit.

## Data Availability

Dragon Fruit Maturity Detection and Quality Grading Dataset (Original data) (Mendeley Data)
